# Molecular Regulation of Nitrate Responses in Plants

**DOI:** 10.3390/ijms19072039

**Published:** 2018-07-13

**Authors:** Lufei Zhao, Fei Liu, Nigel M. Crawford, Yong Wang

**Affiliations:** 1 State Key Laboratory of Crop Biology, College of Life Sciences, Shandong Agricultural University, Tai’an 271018, China; lufeizhao@163.com (L.Z.); liufei280429@163.com (F.L.); 2 College of Agronomy, Liaocheng University, Liaocheng 252059, China; 3 Section of Cell and Developmental Biology, Division of Biological Sciences, University of California at San Diego, La Jolla, CA 92093-0116, USA; ncrawford@ucsd.edu

**Keywords:** *Arabidopsis*, primary nitrate response, long-term nitrate response, nitrate regulation, root development

## Abstract

Nitrogen is an essential macronutrient that affects plant growth and development. Improving the nitrogen use efficiency of crops is of great importance for the economic and environmental sustainability of agriculture. Nitrate (NO_3_^−^) is a major form of nitrogen absorbed by most crops and also serves as a vital signaling molecule. Research has identified key molecular components in nitrate signaling mainly by employing forward and reverse genetics as well as systems biology. In this review, we focus on advances in the characterization of genes involved in primary nitrate responses as well as the long-term effects of nitrate, especially in terms of how nitrate regulates root development.

## 1. Introduction

Nitrogen is an essential macronutrient for plant growth and development and most terrestrial plants absorb nitrate as their main nitrogen source. In agricultural systems, nitrate supply directly affects plant growth and crop productivity [1,2]. In many developed and developing countries, excessive nitrogen fertilizer is applied in agriculture, while the nitrogen use efficiency (NUE) of crops is low. Therefore, a large fraction of the applied nitrogen cannot be taken up by plants and is lost into the environment, resulting in serious problems such as eutrophication and nitrate pollution of underground water. These problems must be addressed. One approach is to improve the NUE of crops, which could reduce the load of nitrogen fertilizers on farm land and natural ecosystems. Elucidating the mechanisms and the underlying network of nitrate regulation would provide a theoretical basis and guiding framework for improving NUE.

Nitrate is absorbed from the external environment into the roots by nitrate transporters (NRT1s and NRT2s). A part of the nitrate imported into cells is reduced and assimilated into amino acid through a series of enzymes including nitrate reductase (NR), nitrite reductase (NiR), glutamine synthase (GS), and glutamate synthase (GOGAT). Nitrate acts as a nutrient and as an important signal to regulate gene expression, plant growth, and development [3]. Nitrate signaling can be divided into short-term and long-term effects. The short-term effect is referred to as the primary nitrate response, in view of the fact that many genes can be regulated after a short period of exposure to nitrate inputs. Indeed, some genes involved in nitrate transport (*NRTs*) and reduction (*NIAs* and *NiR*) are induced in a matter of minutes [4,5,6,7,8]. The long-term effects include the impact of nitrate on plant growth and development after a longer period of time, including effects on the morphogenesis of roots, plant flowering, seed dormancy, stomatal closure independent of abscisic acid, the circadian rhythm, and the transport of auxin [9,10,11,12,13,14]. Among these aspects, the effects of nitrate on root development are well studied and several essential genes involved in this process have been identified. Here we review the genes involved in the primary nitrate response and describe their functions in nitrate signaling (Table 1). Then we summarize the relationship between nitrate availability and root system architecture and the roles of the characterized genes that control root growth and development in response to local and systemic nitrate signals (Table 1).

## 2. Short-Term Nitrate Signaling: The Primary Nitrate Response

In the late 1990s, molecular components involved in nitrate signaling were identified in bacteria and fungi [15]. In *Escherichia coli*, both NARX and NARQ containing a P-box domain were found to be responsible for nitrate binding and could activate the nitrate-regulating proteins NarL and NarP, which are essential for nitrate sensing. Therefore, these two genes are nitrate regulators in *E. coli* [15,16,17]. In fungi, two transcription factors NirA and Nit4 have been identified as important nitrate regulators. NirA is needed for the expression of nitrate reductase and Nit4 may interact with nitrate reductase directly. Both proteins were demonstrated to activate their target genes that can respond to nitrate [15,18,19,20].

In plants, some genes encoding proteins required for nitrate assimilation, transport, and energy and carbon metabolism are rapidly induced after nitrate treatment [6,8,10]. These are regarded as primary nitrate-responsive genes. Scientists have characterized a few of the regulators playing important roles in primary nitrate responses, mainly by employing methodologies in forward and reverse genetics as well as systems biology.

### 2.1. Nitrate Sensor

NRT1.1, also called CHL1 and NPF6.3, belongs to the NRT1/PTR family (NPF) [15,21]. Previously, NRT1.1 was identified as a dual-affinity nitrate transporter working in both low and high nitrate concentrations [22,23,24,25]. Subsequently, it was shown that NRT1.1 controlled root architecture by acting as a potential nitrate sensor [26,27]. Then in 2009 it was found that NRT1.1 is involved in the primary nitrate response [28,29]. Using a forward genetic screen, the Crawford lab identified a mutant with a defective response to nitrate, and the mutation was localized to *NRT1.1* [29]. Characterization of the mutant revealed that expression of the primary nitrate-responsive genes *NIA1*, *NiR*, and *NRT2.1* was significantly inhibited when plants were grown in the presence of ammonium. Interestingly, the regulatory role of *NRT1.1* was lost when ammonium was absent because the expression of these nitrate-responsive genes was restored in the mutant without ammonium [29], indicating that other nitrate sensor(s) were present and dominated in the absence of ammonium. The Tsay lab also showed that a null mutant of *NRT1.1*, *chl1-5,* lost both nitrate uptake and primary nitrate response functions [28]. They then described an allele of *NRT1.1* (*chl1-9*) that was defective in nitrate uptake but not nitrate regulation. These results indicate that the primary nitrate response was defective in the mutant *chl1-5* but not in *chl1-9*, and the function of *NRT1.1* in nitrate signaling is independent of its uptake activity, thereby identifying NRT1.1 as a nitrate sensor [28]. This research also found that when *NRT1.1* was phosphorylated at a low nitrate concentration, it was involved in maintaining the low-level primary response; when it was dephosphorylated under a high nitrate concentration, it led to a high-level primary response [28]. 

More recent work has shown that *NRT1.1*-mediated regulation is quite complex in that it activates distinct signaling mechanisms (see below for more details) [30]. Furthermore, a rice homolog of *AtNRT1.1 (OsNRT1.1B)* has been identified, and variations in this gene in the rice (*Oryza sativa*) sub-species *indica* have been identified as boosting the absorption of nitrate and the transport of nitrate from roots to shoots, and potentially enhance NUE in rice [31].

### 2.2. Transcription Factors

Another important nitrate regulator is the transcription factor NLP7, which belongs to the NIN (nodule inception protein)-like protein family in *Arabidopsis*. The NIN protein family was originally found to function in the initiation of nodule development in legume species and these family members are conserved in higher plants and algae [32,33,34,35]. The NIT2 protein is a homologue of the NIN family in *Chlamydomonas* and can bind to the promoter of the nitrate reductase gene [35,36]. In *Arabidopsis*, NLP7 has been demonstrated to be an important positive regulator of primary nitrate response as the induction of the nitrate-responsive genes *NIA1*, *NIA2*, *NRT2.1*, and *NRT2.2* is inhibited and nitrate assimilation is also impaired in *nlp7* mutants [35,37]. 

The function of NLP7 in nitrate signaling was further confirmed by the identification of the *nlp7* mutant in an effort to explore novel nitrate regulators by using a forward genetics approach [29]. ChIP-chip analysis revealed that NLP7 could bind 851 genes including genes involved in N metabolism and nitrate signaling, such as *NRT1.1*, *CIPK8*, *LBD37/38*, and *NRT2.1* [38]. A recent study found that NLP7 could regulate the expression of *NRT1.1* in the presence of ammonium and bind directly to the promoter of *NRT1.1*. These findings illustrate that *NLP7* works upstream of *NRT1.1* directly when ammonium is present [39]. NLP7 can also activate or repress nitrate-responsive genes [38,40]. 

The *Arabidopsis thaliana* genome encodes nine NLPs, all of which contain the conserved RWP-RK domain and the PB1 domain. Members of this family can be divided into four subgroups: NLP1 and 2, NLP4 and 5, NLP6 and 7, and NLP8 and 9 [34,35]. Yeast one-hybrid (Y1H) screening using four copies of the nitrate response *cis*-element (NRE) illustrated that all NLPs could bind to the NRE element [41]. In response to nitrate, the transcript levels of *NLP* genes are not regulated, but examination of an NLP7-green fluorescent protein (GFP) fusion revealed that localization of NLP7 was modulated by nitrate via a nuclear retention mechanism [38,41]. Recently, this localization of NLP7 was identified to occur when Ser205 in NLP7 was phosphorylated in vivo in the presence of nitrate [42]. 

Suppression of the NLP6 function resulted in the downregulation of nitrate-responsive genes, indicating that NLP6 is also a master nitrate regulatory gene involved in primary response [41]. Further characterization has shown that the N-terminal region of NLP6 is necessary for its activation in response to nitrate signaling [41]. Furthermore, using overexpression lines, *NLP7* was revealed to significantly improve plant growth under nitrogen-poor and -rich conditions [37]. Moreover, *ZmNLP4* and *ZmNLP8*, maize homologs of *AtNLP7*, play essential roles in nitrate signaling and assimilation and promote plant growth and yield under low nitrate conditions, implying that they may be potential candidates for improving the NUE of maize (*Zea mays*) [43].

In addition to NLPs, reverse genetics has identified LBD37/38/39 to be important nitrate regulators [44,45]. *LBD37/38/39* belong to a gene family encoding zinc-finger DNA binding transcription factors and are strongly induced by nitrate. Further characterization revealed that overexpression of *LBD37/38/39* can repress the expression of nitrate-responsive genes including *NRT2.1*, *NRT2.2*, *NIA1*, and *NIA2*, indicating that the three LBD members function as negative regulators in nitrate signaling [44]. 

Recently, following advances in bioinformatics and global sequencing analysis, systems biology approaches have been developed and successfully applied to plant nitrogen research. The transcription factors SPL9, TGA1, and TGA4 have been sequentially identified by systems approaches. SPL9 was predicted to be a potential regulatory hub and may target sentinel primary nitrate-responsive genes [46]. Research has demonstrated that miR156 can target *SPL9* and a mutation in the miR156 caused overexpression of *SPL9* [47]. Accordingly, miR156-resistant SPL9 transgenic plants (the rSPL9 mRNA resulting from the modified gene is resistant to degradation by miR156) were investigated and it was revealed that the transcript levels of *NRT1.1*, *NIA2*, and *NIR* significantly increased in response to nitrate, demonstrating that *SPL9* plays a negative role in the primary nitrate response [46].

TGA1 and TGA4 belong to the bZIP transcription factor family and are induced by nitrate in roots [48]. Interestingly, induction of *TGA1* and *TGA4* is inhibited in *chl1-5* and *chl1-9* mutants after nitrate treatment, implying that the regulation of *TGA1* and *TGA4* by nitrate is affected by nitrate transport, but not the signaling function of *NRT1.1* [48]. Transcriptome analysis of the roots of *tga1 tga4* double mutant plants revealed that most of the genes differentially expressed in the double mutant were regulated by nitrate. Among these target genes of TGA1 and TGA4, induction of *NRT2.1* and *NRT2.2* was substantially reduced in the double mutants. Further analysis demonstrated that TGA1 could bind to *NRT2.1* and *NRT2.2* promoters to positively regulate their expression [48]. These results all serve to suggest that TGA1 and TGA4 play important roles in the primary nitrate response.

Recently, Shuichi’s lab found that nitrate-inducible GARP-type transcriptional repressor1 proteins (*NIGT1s*) act as central regulators in nitrate signaling [49]. Co-transfection assays revealed that NIGT1-clade genes including *NIGT1.1/HHO3*, *NIGT1.2/HHO2*, *NIGT1.3/HHO1,* and *NIGT1.4/HRS1* were all induced by nitrate and were redundant in repressing the nitrate-dependent activation of *NRT2.1*. EMSA and chromatin immunoprecipitation–quantitative PCR (ChIP-qPCR) analysis further showed that NIGT1.1 could directly bind to the promoter of *NRT2.1* [49]. Transcriptome and co-transfection analysis also illustrated that the expression of *NIGT1s* was autoregulated and controlled by *NLPs*. In addition, *NIGT1.1* can suppress the activation of *NRT2.1* by NLP7 [49]. Further investigation suggested that the regulation of *NRT2.1* by NIGT1.1 and NLP7 is independent due to their distinct binding sites. A genome-wide survey discovered the potential target genes that might be regulated by both *NLP*-mediated activation and *NLP*-*NIGT1* transcriptional cascade-mediated repression or the *NLP*-*NIGT1* cascade alone [49]. Furthermore, phosphate starvation response 1 (PHR1), the master regulator of P-starvation response, also directly enhanced the expression of *NIGT1*-clade genes, serving to reduce nitrate uptake [49,50]. 

### 2.3. Protein Kinases

CIPK8 and CIPK23 are calcineurin B-like (CBL)-interacting protein kinases. *CIPK8* is induced rapidly by nitrate and downregulated in the *chl1-5* mutant. Analysis of two independent T-DNA insertion lines (*cipk8-1* and *cipk8-2*) showed that induction of *NRT1.1*, *NRT2.1*, *NIA1*, *NIA2*, and *NiR* by nitrate was reduced in *cipk8* mutants indicating that CIPK8 works as a positive regulator in the primary nitrate response [51]. Further investigation revealed that *CIPK8* regulated the nitrate-induced expression of *NRT1.1* and *NRT2.1* under higher (25 mM) but not lower nitrate conditions (250 µM), suggesting that CIPK8 functions as a positive regulator when nitrate is replete [3,51]. CIPK23 can be induced by nitrate and downregulated in the *chl1-5* mutant like *CIPK8* [28]. Expression of the nitrate responsive gene *NRT2.1* was upregulated in the *cipk23* mutants after nitrate treatment, indicating that *CIPK23* plays a negative role in primary nitrate response [28]. This gene is essential for the affinity switch of NRT1.1: it interacts with NRT1.1 and phosphorylates NRT1.1 at T101 under low nitrate concentrations to enable NRT1.1 to operate as a high affinity nitrate transporter, while it dephosphorylates NRT1.1 when nitrate is replete so that NRT1.1 functions as a low-affinity nitrate transporter [28].

CPK10, CPK30, and CPK32 are subgroup III Ca^2+^-sensor protein kinases (CPKs). The activity of CPKs can be enhanced in response to nitrate within 10 min. They have all been identified as master regulators that orchestrate primary nitrate responses [42]. Analysis of the single *cpk10*, *cpk30*, and *cpk32* mutants has shown that they only trivially affect nitrate-responsive genes. However, in the double mutants *cpk10 cpk30*, *cpk30 cpk32*, and *cpk10 cpk32* and the triple mutant *cpk10 cpk30 cpk32*, nitrate-responsive marker genes were reduced. Transcriptomic analysis showed that CPK10, CPK30, and CPK32 modulated various key cellular and metabolic functions immediately activated by nitrate. Furthermore, CPK10, CPK30, and CPK32 can phosphorylate NLP7 at Ser205 in vivo in the presence of nitrate, and trigger the nitrate-CPK-NLP signaling network [42].

### 2.4. Other Factors, Including NRG2, CPSF30, and FIP1

Recently, three other nitrate regulatory genes *NRG2*, *CPSF30-L*, and *FIP1* were identified using a forward genetics method [52,53,54]. Two independent *NRG2* T-DNA insertion lines (*nrg2-1* and *nrg2-2*) showed reduced induction for nitrate-responsive sentinel genes (*NIA1*, *NIR*, *NRT2.1*), indicating that *NRG2* plays an essential role in nitrate signaling. At the physiological level, *NRG2* affects accumulation of nitrate in plants. Further investigation revealed that it regulates nitrate uptake by roots and the translocation of nitrate within plants. These effects might be achieved through modulating *NRT1.1* and *NRT1.8* as the expression of both genes was altered in the mutants [52]. Genetic and molecular data suggest that *NRG2* can regulate the expression and work upstream of *NRT1.1*, but function independently, with *NLP7* in regulating nitrate signaling. In addition, transcriptomic analysis showed that four clusters in the differentially expressed genes in *nrg2* mutant were involved in the regulation of nitrate transport and response, confirming that *NRG2* plays essential roles in nitrate regulation. Interestingly, NRG2 can directly interact with NLP7 in vitro and in vivo, as revealed by yeast two hybrid and BiFC experiments [52]. All these results demonstrate that NRG2 is an important nitrate regulator. 

In addition, the *Arabidopsis* genome harbors 15 members that are homologous with the NRG2 protein. All members of the NRG2 family contain two unknown conserved domains: DUF630 and DUF632. Whether and which other members of the NRG2 family are involved in nitrate signaling and what functions the two domains play are interesting and pertinent directions for future research. 

The *CPSF30* gene encodes 28-kD and 65-kD proteins. The 28-kD protein (CPSF30-S) was identified as a cleavage and polyadenylation specificity factor [53,55,56,57]; the protein contains three characteristic CCCH zinc finger motifs and functions as both an endonuclease and an RNA-binding protein [56,58]. An additional YTH domain, along with the three zinc finger motifs, are contained in the 65-kD protein (CPSF30-L) [53]. A mutant allele of *CPSF30*, *cpsf30-2* with a G-to-A mutation in the first exon of gene *CPSF30*, was identified by genetic screening and used to explore the functions of *CPSF30* [53]. The expression of nitrate-responsive genes (*NIA1*, *NiR*, *NRT2.1*) can be downregulated in response to nitrate in *cpsf30-2* compared to wild-type and restored to wild-type levels in a complemented *CPSF30-L/cpsf30-2* line, indicating that *CPSF30-L* is involved in nitrate signaling. *CPSF30-L* can regulate nitrate accumulation and assimilation at the physiological level [53]. Transcriptomic analysis showed that genes involved in six nitrogen-related clusters, including nitrate transport and assimilation, were differentially expressed in the *cpsf30-2* mutant. Further study revealed that *CPSF30* could work upstream of *NRT1.1* and independently of *NLP7*. *CPSF30* can also affect *NRT1.1* mRNA 3′UTR alternative polyadenylation [53]. All these results demonstrate that CPSF30 plays an important role in the primary nitrate response.

FIP1, a factor interacting with poly(A) polymerase 1, was identified as a positive nitrate regulatory gene using the *fip1* mutant and a *FIP1/fip1* line [54]. Nitrate-induced expression of *NIA1*, *NiR*, and *NRT2.1* is repressed in the *fip1* mutant and can be restored to the wild type in the *FIP1/fip1* line [54]. Furthermore, *FIP1* can affect nitrate accumulation through regulating the expression of *NRT1.8* and nitrate assimilation genes [54]. Further research found that FIP1 could interact with CPSF30 and both genes can regulate the expression of *CIPK8* and *CIPK23* [54]. In addition, *FIP1* can affect the 3′UTR polyadenylation of *NRT1.1*, a similar function to *CPSF30* [54]. CPSF30, FIP1, and some other components such as CPSF100 can form a complex involved in poly (A) processing [59]. Together, these findings suggest that the complex composed by CPSF30 and FIP1 may play important roles in nitrate signaling.

**Figure 1:**
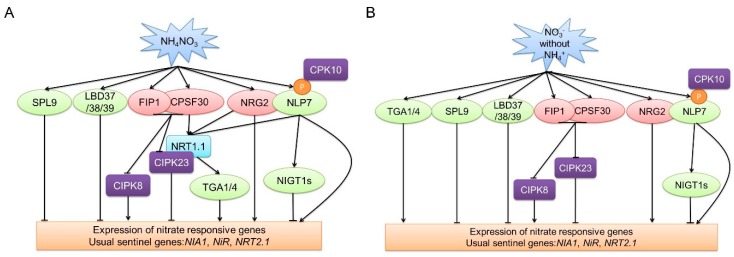
Schematic representation of nitrate regulatory factors affecting nitrate signaling. (**A**) The schematic for nitrate regulatory factors in the present of both ammonium and nitrate; (**B**) the schematic for nitrate regulatory factors without ammonium. The blue box indicates a nitrate sensor. Light green boxes indicate transcription factors. Purple boxes indicate protein kinases. Light red boxes indicate other nitrate signaling regulators. Arrows indicate positive regulation. Blunted lines indicate negative regulation.

In the extant literature, key molecular components involved in primary nitrate responses, covering nitrate sensors, transcription factors, protein kinases, and polyadenylation specificity factors, have been identified. Methodologically, this has been achieved by using forward and reverse genetics as well as systems biology approaches (Table 1). In summary, in the presence of both ammonium and nitrate (Figure 1A), NRT1.1 functions as a sensor. *NLP7*, *NRG2*, and *CPSF30* have been revealed to work upstream of *NRT1.1* [39,52,53]. NRG2 can interact with NLP7 [52] whilst NLP7 can interact with, and be phosphorylated by, CPK10 [42]. In addition, NLP7 binds to the promoter of *NRT1.1* as revealed by ChIP and EMSA assays [39]. *NRT1.1* works upstream of, and regulates, *TGA1/TGA4* [60]. Furthermore, CIPK23 interacts with and phosphorylates NRT1.1 [28]. CPSF30 can interact with FIP1 and regulate the expression of both *CIPK8* and *CIPK23* [54]. *NIGT1.1* can suppress NLP7-activated *NRT2.1* [49]. In the presence of nitrate but absence of ammonium (Figure 1B), NRT1.1 works only as a nitrate transporter, but not as a nitrate regulator. The other nitrate regulatory genes, including *NRG2*, *NLP7*, *CPSF30*, *FIP1*, *LBD37/38/39*, *SPL9*, *NIGT1s*, *CIPK8*, and *CIPK23*, still play an important role in the nitrate signaling. 

## 3. Long-Term Nitrate Signaling

Serving as an important molecular signal, nitrate also regulates plant growth and development and has been particularly well studied in the context of root system architecture. Root system architecture controls the absorption and utilization of nutrients and affects the growth and biomass of plants. Lateral root growth is dually regulated by nitrate availability, including local induction by NO_3_^−^ and systemic repression by high NO_3_^−^ [61,62,63]. Several key genes and miRNAs functioning in nitrate-regulated root architecture have been characterized.

The *ANR1* gene, encoding a member of the MADS-box family of transcription factors, was the first gene to be identified as an essential component in nitrate-regulated root growth [51]. Nitrate can inhibit the growth of lateral roots when seedlings are grown on media with higher nitrate concentrations compared to lower nitrate concentrations (≤1 mM). However, *ANR1* downregulated lines obtained by antisense or co-suppression exhibited reduced lateral root length when grown on media with various nitrate concentrations, indicating the enhanced sensitivity of lateral root growth to nitrate inhibition in those lines [61]. Overexpression of *ANR1* in roots resulted in increased lateral root growth and this phenotype was strongly dependent on the presence of nitrate, suggesting posttranslational control of ANR1 activity by nitrate [45,64]. Interestingly, the expression of *ANR1* in *nrt1.1* mutants was dramatically diminished and these mutants exhibited reduced root elongation in nitrate-rich patches, similar to what was observed with the *ANR1*-repressed lines [26,61]. This suggests that *NRT1.1* works upstream of *ANR1* in terms of local nitrate-induced lateral root growth. Recently, the auxin transport role of *NRT1.1* was characterized in lateral root primordia (LRPs) when seedlings were grown on media without nitrate or with low nitrate concentrations; under these conditions, *NRT1.1* represses the growth of pre-emerged LR primordia and young LRs by inhibiting the accumulation of auxin [14]. Subsequently, Gojon’s lab revealed that the *NRT1.1*-mediated regulation of LR growth was dependent on the phosphorylation of NRT1.1 and the non-phosphorylated form of NRT1.1 could transport auxin in the absence of nitrate or in low nitrate concentrations [30]. Further investigation indicated that in the presence of nitrate, the promoter activity of *NRT1.1* was stimulated and mRNA stability was increased, while protein accumulation and auxin transport activity were repressed in LRPs, resulting in accelerated lateral root growth [65]. Altogether, NRT1.1 offers a link between nitrate and auxin signaling during lateral root development. However, the mechanisms by which nitrate induces the expression of *NRT1.1* while repressing NRT1.1 protein accumulation and auxin transport activity in LRPs remain unclear. Previous reports have also documented that several genes involved in hormone biosynthesis or response regulate the root system architecture response to changes in nitrate availability [66,67].

NRT2.1, a high-affinity nitrate transport gene, is induced by nitrate and sugar [68,69,70,71]. Wild-type seedlings grown on media with high carbon/nitrogen (C/N) ratios exhibited significantly repressed lateral root initiation compared to a standard growth medium [72]. However, the repression of lateral root initiation was diminished in *nrt2.1* mutants under high C/N ratios where this phenotype was not dependent on nitrate uptake [63,73]. These results demonstrate that NRT2.1 plays an important role in lateral root initiation under high C/N ratios. In addition, *nrt2.1* mutants exhibited significantly reduced shoot-to-root ratios compared to wild-type and *nrt2.2* mutant seedlings when grown in common hydroponic conditions (grown on 1 mM NH_4_NO_3_ for four weeks followed by one-week nitrogen starvation). The reductions in shoot-to-root ratios were even greater for *nrt2.1 nrt2.2*, suggesting that both genes are involved in regulating plant growth with NRT2.1 playing a more important role [74]. Moreover, *nrt2.1* mutants exhibit reduced LR growth on media with limited nitrogen [63,74] and this reduction was more severe in *nrt2.1 nrt2.2* double mutant plants, indicating that both genes are important regulators involved in lateral root growth [74]. Recently, Gutierrez’s lab determined that induction of *NRT2.1* and *NRT2.2* was directly regulated by TGA1/TGA4 in response to nitrate treatment. Further investigation showed that *tga1 tga4* plants and *nrt2.1 nrt2.2* plants exhibited similarly decreased LR initiation compared with wild-type plants, indicating that *NRT2.1* and *NRT2.2* work downstream of *TGA1/TGA4* to modulate LR initiation in response to nitrate. Lateral root emergence was also affected in *tga1 tga4* and *nrt2.1 nrt2.2* mutants, and *tga1 tga4* mutants displayed larger reductions in LR emergence than *nrt2.1 nrt2.2* mutants, revealing that additional pathways are required for LR emergence controlled by TGA1/TGA4 besides *NRT2.1* and *NRT2.2*. Moreover, primary roots in *tga1 tga4* mutants were shorter than in wild-type and *nrt2.1 nrt2.2* plants, suggesting that the modulation of primary root growth by TGA1/TGA4 is independent of *NRT2.1* and *NRT2.2* [48]. 

The protein kinase CIPK8 is not only involved in primary nitrate response, but also in long-term nitrate regulation on root growth. In the presence of nitrate, *cipk8* mutants exhibited longer primary root length compared to the wild type, indicating that *CIPK8* modulates primary root growth in a nitrate-dependent pathway [51]. Furthermore, the key nitrate regulator NLP7 has also been found to control root growth under both N-limited and N-rich conditions besides its essential roles in the primary nitrate response [35,37]. *nlp7* mutants developed longer primary roots and higher LR density on N-rich media [35]. Interestingly, transgenic lines with overexpression of NLP7 also exhibited increased primary root length and lateral root density under 1, 3, and 10 mM nitrate conditions [37]. The underlying inter-phenotype mechanisms regulating root growth in the mutant and overexpression lines are still unknown. These findings indicate that NLP7 plays an important role in nitrate-regulated root development. Recently, it has been shown that the Ca^2+^-sensor protein kinases CPK10, CPK30, and CPK32 are also involved in nitrate-specific control of root development. In response to nitrate, *icpk* mutants had reduced lateral root primordia density and reduced lateral root elongation compared to the wild type [42].

In the last few years, microRNAs (miRNAs) have emerged as important regulators involved in nitrate-regulated root growth. It has been reported that miR167 targets and controls expression of the auxin response factor ARF8, and both *miR167* and *ARF8* are expressed in the pericycle and lateral root cap [75,76]. Levels of miR167 were repressed under nitrogen treatment, leading to accumulation of ARF8 in the pericycle. In contrast to wild-type plants, which displayed increased ratios of initiating vs. emerging lateral roots in response to nitrogen treatment, the *miR167a* overexpression lines and *arf8* mutants were insensitive to nitrogen in terms of lateral root emergence. These results indicate that the auxin response factor-miRNA regulatory module miR167/*ARF8* plays an important role in controlling lateral root growth in response to nitrogen [75]. In addition, *miR393* was induced by nitrate treatment, specifically cleaved the auxin receptor *AFB3* transcript, and modulated the accumulation of *AFB3* mRNA in roots under nitrate treatment [77]. The primary root of the wild type was shorter when treated with KNO_3_ compared to KCL, however the primary root of the *miR393*-overexpression line and *afb3* mutant were insensitive to nitrate treatments. miR393/*AFB3* also controlled lateral root growth as well as primary root growth. The miR393 overexpression line and *afb3* mutant showed diminished densities of initiating and emerging lateral roots compared to the wild type, which exhibited increased growth of lateral roots in response to nitrate treatments. Further investigation found that transcription factor NAC4 acted downstream of AFB3 to regulate lateral root growth in response to nitrate, but did not affect primary root growth, indicating that AFB3 is likely to be involved in two dependent pathways to modulate root system architecture. Furthermore, *AFB3* and *NAC4* gene expression in response to nitrate treatment depends on the nitrate transport function of *NRT1.1* [78,79]. Moreover, peptide-mediated signaling has been found in N control of root growth. The Arabidopsis *CLE* gene was found to be induced by N-deficiency, and overexpression of *CLE* inhibits lateral root elongation but not initiation [80]. The peptide sequence of CLE is homologous to CLV3, which binds to *CLV1* and the *clv1* mutant showed increased lateral root length under low N conditions. The transcript levels of *CLE* were increased in the *clv1* mutant, suggesting a feedback regulation of *CLE* by *CLV1*. Transgenic lines with increased CLE levels in *clv1* did not inhibit lateral root growth, indicating that the inhibition of *CLE3* on lateral root development requires CLV1. Altogether, the N-responsive CLE-CLV1 peptide-receptor signaling module restricts expansion of the lateral root system in N-deficient environments [80].

Although nitrate is a crucial nutrient and signaling molecule, its distribution in soils is heterogeneous. To adapt the prevailing nitrate conditions, plants have evolved a systemic response mechanism. NRT2.1 was the first molecular target identified in long-distance signaling reflecting root responses to environmental nitrate conditions [81]. Plants were grown using a 1 mM NO_3_^−^ solution, then the root was split into two parts, one subjected to N-free treatment and the other one treated with 1 mM NO_3_^−^. Both ^15^NO_3_^−^ influx and the transcript level of *NRT2.1* were increased in the NO_3_^−^-fed root. 

Recent findings revealed that the C-terminally encoded peptide (CEP) originated from N-starved roots; located in xylem vessels, it sends root-derived ascending signals to the shoot before being recognized by a leucine-rich repeat receptor kinase, CEPReceptor 1 (CEPR1), and then inducing the expression of CEPD polypeptides. CEPD sent long-distance mobile signals translocated to each root and upregulated the expression of *NRT2.1* [81,82,83]. 

The activity and expression of *NRT2.1* in plants were inhibited when supplied with high N. Lepetit’s lab configured a forward genetic approach using a transgenic line expressing the pNRT2.1::LUC construct as a reporter gene [84]. The mutant *hni9*, showing increased expression of *NRT2.1* under high N supply, was isolated and the mutation was found in *IWS1*, a component of the RNAPII complexes. Further investigation revealed that the levels of the H3K27me3 on NRT2.1 chromatin decreased, resulting in the upregulated expression of NRT2.1 in response to high N supply in the *iws1* mutants. Thus IWS1 is likely to be involved in the transduction of N systemic signals through controlling the expression of *NRT2.1* in plants [71,84]. 

Another important player participating in root foraging, TCP20, was identified by Crawford’s lab using the yeast one-hybrid system to screen the transcription factors that can bind to the fragment of nitrate enhance DNA [85]. TCP20 was found to be able to bind to the promoters of *NIA1*, *NRT1.1*, and *NRT2.1*. The *tcp20* mutants exhibited deficiencies in preferential lateral root growth on heterogeneous media in split-root experiments, indicating that TCP20 can regulate the preferential growth of lateral roots in high nitrate zones, thus playing an important role in the systemic signaling pathway [85]. Recently, using an electrophoretic mobility shift assay (EMSA), the DNA-binding sites of TCP20 in a 109 bp *NIA1* enhancer fragment were found to be in close proximity to NLP7 and NLP6 binding sites [58]. Yeast two-hybrid and bimolecular fluorescence complementation (BiFC) assays showed that NLP7 and NLP6 can interact with TCP20 and both the PB1 domains of NLP6&7 and the glutamine-rich domain of TCP20 are necessary for protein–protein interaction [58]. Further work will be needed to elucidate the underlying molecular mechanism explaining the involvement of *TCP20* in systemic signaling.

**Figure 2:**
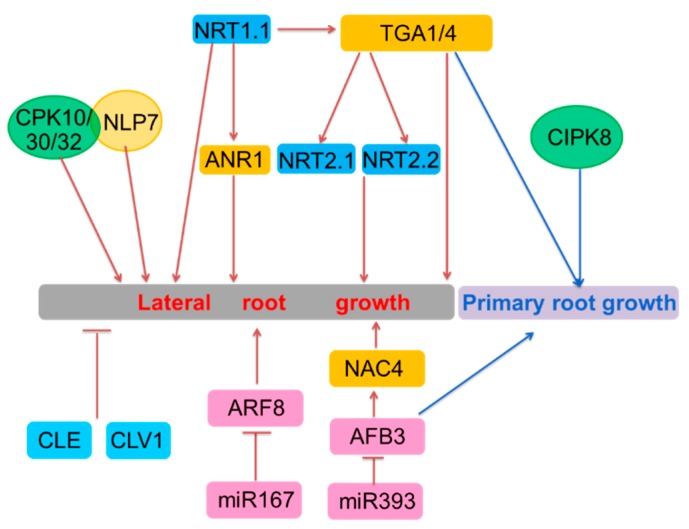
Schematic representation of primary and lateral root growth regulation by nitrate (local signaling). Arrows indicate positive regulation; blunted lines represent negative regulation. The dark blue boxes indicate the nitrate transporters; the yellow boxes indicate the transcription factors; the green boxes indicate the protein kinases; the light blue indicate the peptides; the pick boxes indicate the factors related to auxin.

We proposed a model according to the functions of the genes discussed herein and their relationship in regulating the growth of roots (Figure 2). *NRT1.1* works upstream of *ANR1* and both promote the development of LR [26,61]. Under N limited conditions, NRT1.1 also transports the auxin in LRPs and inhibits the growth of LRs [14,30] (Figure 2). *NRT1.1* works upstream and affects the nitrate induction of *TGA1/4*, but whether NRT1.1 modulates *TGA1/4*-mediated LR is still unknown. *TGA1/4* modulates the development of PR and LR, either independent from or dependent on *NRT2.1* and *NRT2.2* [48,63,74]. *CIPK8* modulates primary root growth in the nitrate-dependent pathway. NLP7 and CPKs also dictate the growth plasticity of roots [35,37,42] (Figure 2). Nitrogen represses *miR167* and induces *miR393*, and both miRNAs regulate the expression of their corresponding target genes and change the architecture of roots in response to nitrogen treatment [75,77] (Figure 2). Moreover, the CLE-CLV1 peptide-receptor signaling module restricts expansion of the lateral root system in N-deficient environments [80] (Figure 2). Although a few genes that function in root growth have been characterized, our understanding of the root system architecture regulation network remains incomplete. Identification of more root system architecture-related genes and deciphering their relationships is still needed to provide the theoretical basis for improving NUE and breeding new crop varieties with high yield.

**Figure 3:**
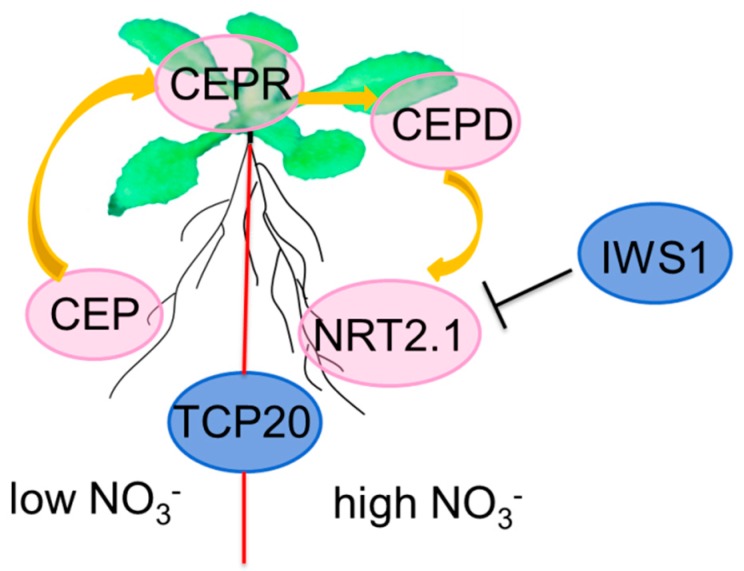
Key genes involved in the systemic signaling pathway that direct nitrate foraging and lateral root growth under heterogeneous conditions. Arrows indicate positive regulation. Blunted lines indicate negative regulation.

Nitrate also acts as a systemic signal to regulate root growth. The distribution of nitrate in soils is often uneven, so plants have evolved complex systemic long-distance signaling mechanisms. Roots in N-starved environments secrete the root-to-shoot mobile peptide hormone CEP, which is perceived by CEPR in shoots. This perception induces CEPD to act as a shoot-to-root secondary signal followed by the increased expression of *NRT2.1* and the growth of LR in nitrate-rich patches [81,82,83]. *IWS1* can repress the expression of *NRT2.1* under high nitrate conditions [71,84]. *TCP20* can modulate the preferential growth of LR in high nitrate zones [85] (Figure 3).

## 4. Conclusions

In the last decade, a number of important genes involved in nitrate signaling have been characterized. In Table 1, we summarize these genes in terms of gene family, target genes, gene functions, and the method of gene discovery. Some of these genes are involved in short-term nitrate signaling, referred to as the primary nitrate response, including *NRT1.1*, *NLP7*, *NLP6*, *LBD37/38/39*, *SPL9*, *TGA1/4*, *NIGT1s*, *CIPK8/23*, *CPK10*, *NRG2*, *CPSF30*, and *FIP1*. Other genes participate in long-term nitrate signaling that affects plant growth and development over a longer time horizon; this is exemplified herein in terms of root architecture, i.e., *ANR1*, *NRT1.1*, *NRT2.1/2.2*, *TGA1/4*, *CIPK8*, *NLP7*, *CPK10*, miR167/*ARF8*, miR393/*AFB3*, *NAC4*, *CLE*-*CLV1*, *CEP*, *HIN9/IWS1*, and *TCP20* (Table 1). Some genes have been identified as being involved in both short-term and long-term nitrate signaling, including *NRT1.1*, *TGA1/4*, *CIPK8*, *NLP7*, and *CPK10* (Table 1). Whether and which other genes are also involved in the short and long term remains unknown. These genes were identified using different approaches, such as forward and reverse genetics, systems biology, and genome-wide analyses. These genes function as sensors, transcription factors, protein kinases, and polyadenylation specificity factors; they affect the primary nitrate response and growth and development of plants both directly and indirectly (Table 1).

Despite the characterization of the above genes involved in nitrate signaling, we are still far from completely understanding the molecular regulation of nitrate signaling in plants. New genes performing essential roles in short-/long-term nitrate effects remain to be identified, which will help us to decipher the regulatory mechanisms of the absorption and utilization of nitrate by plants. With the rapid development of science and technology, some innovated methods used for discovering new nitrate-related genes have been emerged, for example, non-coding RNA analysis, proteomics, and metabolomics. The application of these new technologies will further promote the advancement of nitrate research field. Although relationships among some nitrate regulatory genes have been uncovered, gene networks in plants are still poorly understood and so this is fruitful territory for further research. Recently, the interaction between nitrate and hormonal signaling on controlling the development and stress response of plants has been gradually unraveled. However, the potential crosstalk of nitrate and hormonal signaling is unclear and needed more conscious efforts in the near future. Addressing the pollution problems associated with anthropogenic nitrogen fertilization is also critically important, and thus the NUE of crops must be improved. The research results based on *Arabidopsis* may provide important directions and methodologies for strengthening the NUE study in crops. In the last few years, some homologous genes of *Arabidopsis* have been identified as essential nitrate regulatory genes in crops, such as *ZmNLP4* and *ZmNLP8* in maize and *OsNRT1.1B* in rice. More such genes and their function mechanisms need to be identified to sustain high crop yields and solve the environmental problems.

## Figures and Tables

**Table 1:** Summary of nitrate regulatory genes in the short-term and long-term responses.

The Group of the Genes	Gene	Gene Family	Target Genes	Roles in Nitrate Signaling	Nitrate Responsive	Identification Methodology
Genes involved in short-term nitrate signaling/primary nitrate response	*NLP6*	RWP-PK	*NRT2.1*, *NRT2.2*, and *NIA*	Involved in primary nitrate response (positive)	No	Binding to NRE
	*LBD37/38/39*	LBD	*NRT2.1*, *NRT2.2*, *NIA1*, and *NIA2*	Involved in primary nitrate response (negative)	Yes	Nitrate-responsive transcription factor
	*SPL9*	SPL	*NiR*, *NIA2*, and *NRT1.1* (potential)	Potential regulatory hub (negative)	Yes	Integrated systems biology approach
	*NIGT1s*	NIGT	*NRT2.1*	Involved in primary nitrate response (negative)	Yes	Homologues of *OsNIGT1*
	*CIPK23*	CBL-interacting protein kinases	Phosphorylating NRT1.1	Involved in primary nitrate response (negative)	Yes	Downregulated in *chl1*
	*NRG2*	Nitrate regulatory gene 2	Regulating *NRT1.1* and interacting with NLP7	Involved in primary nitrate response (positive)	No	Forward genetics screening
	*CPSF30*	Polyadenylation specificity factor	Affecting *NRT1.1* mRNA 3’UTR alternative polyadenylation	Involved in primary nitrate response (positive)	No	Forward genetics screening
	*FIP1*	Factor interacting with poly(A) polymerase 1	Interacting with CPSF30, regulating *CIPK8* and *CIPK23*, and affecting 3’UTR alternative polyadenylation of *NRT1.1*	Involved in primary nitrate response (positive)	No	Interaction with CPSF30
Genes involved in both short-term and long-term nitrate signaling	*NRT1.1/NPF6.3*	NPF	Regulating *CIPK8*, *CIPK23*, *TGA1/4*	Involved in primary nitrate response (positive) and regulating lateral root growth	Yes	Forward genetics screening
	*TGA1/4*	bZIP	*NRT2.1* and *NRT2.2*	Involved in primary nitrate response (positive) and regulating lateral root emergency and primary root growth	Yes	Integrated systems biology approach
	*CIPK8*	CBL-interacting protein kinases	Unknown	Involved in primary nitrate response (positive) and regulating primary root growth	Yes	Downregulated in *chl1-5*
	*NLP7*	RWP-PK	*NRT2.1*, *NiR*, *NRT2.2*, and *NIA*	Involved in primary nitrate response (positive) and regulating lateral root density and primary root growth	No	Homologous to NIN protein in legumes and binding to NRE
	*CPK10*	Subgroup III Ca^2+^-sensor protein kinase	Phosphorylating NLP7	Involved in primary nitrate response (positive) and regulating lateral root primordia density and lateral root elongation	Yes	Induced by nitrate
Genes involved in long-term nitrate signaling	*ANR1*	MADS-box	Regulating *NRT1.1*	Regulating lateral root growth under high nitrate	Yes	Isolated in a screen for nitrate-responsive genes in roots
	*NRT2.1*	NPF	Unknown	Regulating lateral root initiation under the conditions of high C/N ratio and lateral root growth under limited nitrogen	Yes	Forward genetics screening
	*ARF8*	Auxin response factors	Unknown	Induced in the pericycle and lateral root cap and marginally repressed in the stele in response to nitrogen	Yes	Cell-specific response to nitrogen
	miR167	microRNA	Regulating the expression of ARF8	Controlling the lateral root growth in response to nitrogen with *ARF8*	Yes	Regulator of ARF8
	miR393	microRNA	Specifically cleaving *AFB3*	miR393/*AFB3* controlled the lateral root growth and primary root growth	Yes	454 sequencing technology
	*AFB3*	Auxin receptor	Unknown	Induced by nitrate and involved in the regulation of nitrate in primary and lateral root growth		Target of miR393
	*NAC4*	NAM/ATAF/CUC	*OBP4*	Regulating lateral roots induction	Yes	Integrated systems biology approach
	*CLE*	CLAVATA3/ESR-related	*CLV1*	Regulating lateral roots elongation	Induced under N-deficiency	Upregulated by N deficiency
	*CLV1*	XI LRR-RLKs	Feedback regulation of *CLE*	Regulating lateral roots emergence and length	unknown	Bound by CLE
	*CEP*	CEP	Unknown	Root-derived ascending signals to the shoot	unknown	Originating from N-starved roots
	*HIN9/IWS1*	Component of RNAPII complexes	*NRT2.1*	Involved in the transduction of N systemic signal	No	Forward genetics Screening
	*TCP20*	TCP	*NRT1.1*, *NIA*, *NRT2.1*, and *NiR*	Regulating lateral root elongation under high nitrate	No	Binding to the promoter of *NIA1* and *NRT2.1*
